# Updating “A nationally representative dataset of 1549 Americans aged 18 to 94 on interest in, experience with, and barriers to cogeneration, defined as working with older and younger people for social good” with data on religious identity and service attendance

**DOI:** 10.1016/j.dib.2026.112961

**Published:** 2026-06-11

**Authors:** Cal J. Halvorsen, Andrew Joung, Stefanie Weiss, Jacob Stolmeier

**Affiliations:** a Brown School at Washington University in St. Louis, One Brookings Drive, St. Louis, MO 63110, USA; b Unit of Occupational Medicine, Karolinska Institute, C6 Institutet för miljömedicin, C6 Arbetsmedicin Falkstedt, 171 77 Stockholm, Sweden; c Department of Economics, University of Michigan, 4330 North Quad, 105 S State St, Ann Arbor, MI 48109, USA; d CoGenerate, P.O. Box 29542, San Francisco, CA 94129, USA; e NORC at the University of Chicago, 55 East Monroe St, 30th Floor, Chicago, IL 60603, USA

**Keywords:** National and community service, Intergenerational, Age diversity, Generation Z, Millennials, Generation X, Baby boomers, Silent generation

## Abstract

This update article presents an expanded version of a nationally representative dataset focused on “cogeneration,” or younger and older people working together for the greater good. While the initial data release detailed interest in and barriers to intergenerational work, this updated version incorporates five previously unreleased variables concerning religious identity (e.g., Protestant, Catholic, agnostic, atheist) and the extent of attendance at religious services. Data for the new religion-focused variables were collected at the same time as the sociodemographic variables previously published as part of this dataset, although the authors did not originally have access to them due to prior data access agreements. This dataset includes respondent answers from a March 2022 survey of 1549 people, aged 18 to 94, who resided in the U.S. and who were a part of the NORC at the University of Chicago AmeriSpeak® Panel. The survey methodology utilized a probability-based sample of the U.S. household population, collecting responses through both online and telephone interviews to ensure broad representation. The core of the dataset remains focused on respondents’ experiences with paid or volunteer work involving individuals at least 25 years older or younger than themselves. It assesses interest in cogenerational efforts, perceived obstacles, and social priorities such as the environment, education, and mental health. The data also explores whether intergenerational cooperation can mitigate societal divisions and examines the nature of respondents' interactions with different age groups outside of their own families. The updated dataset now comprises 188 variables, including 10 string/text variables derived from open-ended responses. The data are available both in Stata (.dta) and CSV formats, supported by two updated codebooks and a NORC methodological report detailing weighting and other information. These new variables offer a unique opportunity for researchers, policymakers, and religious leaders to better understand the intersection of faith and cogenerational social action, informing future program and policy developments.

Specifications Table

**Table:** 

Subject	Social Sciences / Sociology
Specific subject area	Cogenerational work for the greater good (i.e., working with people of different ages on issues they care about). Similar terms: multigenerational, intergenerational.
Type of data	Raw quantitative (numbers with labels) and qualitative (open-ended responses) data, filtered to remove identifying information. Offered in three forms: one Stata (.dta) and two CSV (one dataset with numbers, one dataset with labels) files.
Data collection	Same as in original data article.
Data source location	Institution: Data accessed via respondents who were a part of the NORC at the University of Chicago AmeriSpeak Panel. Country: Respondents lived throughout the U.S., including in each of the U.S. Census Bureau’s four regions and nine divisions
Data accessibility	Repository name: Boston College Dataverse, supported by the Boston College Libraries and hosted by the Harvard Dataverse Network. Direct URL to data: https://doi.org/10.7910/DVN/AIQ87J [1]
Related data article	C.J. Halvorsen, B. Kelley, J. Emerman, S. Weiss, D. Gleicher, J. Stolmeier, M. L, A nationally representative dataset of 1549 Americans aged 18 to 94 on interest in, experience with, and barriers to cogeneration, defined as working with older and younger people for social good, *Data in Brief* 45 (2022) 108,753. https://doi.org/10.1016/j.dib.2022.108753

## 1 Value of the Data

• These new data add five variables related to religious identity and the extent of attendance at religious services to a nationally representative dataset of U.S.-based adults. As a result, users can cross key variables in the original dataset by these new variables (e.g., religious identity by interest in working with people much younger or older than themselves to improve the world around them) as well as include them in multivariable models.
• Program operators, private and public sector leaders, and researchers may benefit from these new data because they can provide information on the relationship between religious identity and religious service attendance and a broad set of prosocial indicators. For example, leaders and members within communities of faith may find these data helpful in increasing multigenerational engagement within these communities.

## 2 Background

The theory of change behind this dataset (and the survey and subsequent dissemination of results to the general public) is that if we reduce age silos, we can engage more of the population – across the life course – in creating better communities [2,3]. This dataset is a product of a long-running academic-practitioner collaboration between the first author and the nonprofit organization, CoGenerate. The organization’s mission is to increase intergenerational solutions to social problems. The third author, who was engaged in the development of the survey that created this dataset and the subsequent release of the results, is based at CoGenerate. After discussions with CoGenerate in 2022, the first author recommended working with NORC at the University of Chicago to finalize the questionnaire and field it through NORC’s AmeriSpeak Panel. Due to CoGenerate’s recent work related to cogenerational initiatives within religious communities in 2025 and 2026, we worked with AmeriSpeak to make the five new variables related to religious identity and engagement public.

## 3 Data Description

For a full description of the data, please see the original article about this dataset [4]. In summary, several files are provided to increase ease of access: two codebooks (one simplified for most users, and one that differentiates between how questions were asked online (CAWI) and by phone (CATI)), and three databases (one .csv file with numbers only, such as “0″ and “1″; one .csv files with labels, such as “no” and “yes”; and one in Stata’s .dta format, complete with numbers and labels). These files are available for download online [1].

Table 1 includes the sample characteristics. The five new variables are listed at the top of Table 1, and several of sociodemographic variables previously released in the original article are listed below them. Among the new variables, three relate to religious identity: one is the 14-category original variable, and two more are condensed versions (five and two categories) for users’ convenience. Two variables relate to religious service attendance: one is the nine-category original variable, and the second is a condensed, four-category version for users’ convenience.

**Table 1:** Sample characteristics (*N* = 1549).

	Unweighted	Weighted
*New variables*		
**Religious identity**		
Protestant	29.80%	29.04%
Catholic	18.32%	16.62%
Mormon	1.95%	1.83%
Orthodox	0.16%	0.44%
Jewish	1.79%	1.60%
Muslim	0.81%	1.71%
Buddhist	0.49%	0.52%
Hindu	0.90%	1.76%
Atheist	4.97%	4.62%
Agnostic	5.29%	6.08%
Nothing in particular	14.41%	14.76%
Just Christian	17.26%	16.61%
Unitarian	0.33%	0.15%
Something else	3.50%	4.26%
**Religious identity, condensed**^a^		
Protestant	29.80%	29.04%
Catholic	18.32%	16.62%
Other Christian	19.38%	18.88%
Other religion	7.82%	10.01%
Not religious	24.67%	25.46%
**Identifies with a religion**^a^		
Yes	75.33%	74.54%
No	24.67%	25.46%
**Religious service attendance**		
Never	24.16%	26.08%
Less than once per year	14.61%	13.19%
About once or twice a year	12.63%	12.97%
Several times a year	13.88%	14.32%
About once a month	2.79%	2.30%
2–3 times a month	4.63%	3.95%
Nearly every week	7.86%	7.50%
Every week	14.39%	15.16%
Several times a week	5.07%	4.54%
**Religious service attendance, condensed**^b^		
Never	24.16%	26.08%
Rarely (Once a year or less)	14.61%	13.19%
Sometimes (Several times a year)	26.51%	27.28%
Regularly (Once a month or more)	34.73%	33.45%
*Previously published variables*		
**Gender**		
Male	47.97%	48.49%
Female	52.03%	51.51%
**Age**		
18–29	14.46%	20.29%
30–44	26.60%	25.75%
45–59	23.31%	23.60%
60+	35.64%	30.36%
**Race/ethnicity**		
White, non-Hispanic	65.72%	62.54%
Black, non-Hispanic	11.43%	11.98%
Asian, non-Hispanic	3.10%	6.04%
Another race, non-Hispanic	1.48%	0.92%
2+ races, non-Hispanic	2.58%	1.64%
Hispanic	15.69%	16.87%
**Education**		
< High school diploma	4.65%	9.60%
High school graduate or equivalent	17.43%	28.31%
Vocational, technical school, some college, associate’s degree	40.09%	27.07%
Bachelor’s degree	21.43%	18.18%
Post graduate study, professional degree	16.40%	16.83%
**Marital status**		
Married	50.48%	49.80%
Widowed	4.26%	3.04%
Divorced	11.30%	9.78%
Separated	4.13%	4.18%
Never married	23.95%	27.52%
Living with partner	5.87%	5.68%
**Employment status**		
Working (paid employee)	51.45%	50.89%
Working (self-employed)	6.97%	7.74%
Not working (temporarily laid off)	2.19%	1.96%
Not working (looking for work)	3.62%	4.65%
Not working (retired)	22.85%	19.75%
Not working (disabled)	6.52%	7.06%
Not working (other)	6.39%	7.95%
**Household income**		
< $10,000	4.58%	4.70%
$10,000 to < $20,000	8.59%	8.76%
$20,000 to < $30,000	10.26%	10.03%
$30,000 to < $40,000	8.72%	7.60%
$40,000 to < $50,000	9.88%	9.05%
$50,000 to < $75,000	19.43%	19.06%
$75,000 to < $100,000	13.94%	12.80%
$100,000 to < $150,000	15.69%	17.47%
$150,000+	8.91%	10.52%
**U.S. Census division**		
New England	5.23%	4.68%
Middle Atlantic	10.52%	12.51%
East North Central	18.66%	14.23%
West North Central	9.94%	6.40%
South Atlantic	19.17%	20.46%
East South Central	5.42%	5.86%
West South Central	7.94%	11.95%
Mountain	8.97%	7.63%
Pacific	14.14%	16.29%
**Metropolitan area**		
Metro area	84.64%	84.45%
Non-metro area	15.36%	15.55%
**Housing type**		
Owned	66.95%	70.84%
Rented	30.34%	26.90%
Occupied without payment	2.71%	2.26%
**Household size**^c^		
1	18.01%	15.99%
2	35.51%	33.21%
3	15.88%	15.92%
4	13.04%	13.22%
5	9.68%	11.21%
6+	7.88%	10.46%
**Device**		
Computer^d^	40.41%	37.43%
Smartphone	53.65%	56.80%
Tablet	1.23%	1.03%
Phone interview	4.52%	4.49%

a Derived from the “Religious affiliation” variable for users’ convenience.

b Derived from the “Religious service attendance” variable for users’ convenience.

c The number of people who live in the household for at least three months per year, including the respondent.

d Includes desktop and laptop computers.

As explained in the original article about this dataset [4], the survey was designed to understand respondents’ attitudes about, experiences in, and interest in civic engagement activities (e.g., volunteering), particularly with people of different generations. The survey also included more general questions about the value of working together across the generations. Descriptive analysis of some of the survey’s findings can be found in a publicly released report by the lead organization behind the survey, CoGenerate (which was then called Encore.org) [3]. The dataset also contains weights to make the survey nationally representative.

At the request of NORC, we have also dropped six variables from our originally published dataset to reduce the risk of respondent disclosure: the number of household members (separate variables for the following age groups: ages 0–1, 2–5, 6–12, 13–17, and 18+), and respondent state of residence. However, this dataset still contains variables related to total number of members in the household as well as U.S. Census regions (separate variables for the four- and nine-category regions). These should be sufficient to answer most researcher questions.

## 4 Experimental Design, Materials and Methods

As explained in the original data article [4], this survey was designed through a collaboration between CoGenerate, NORC at the University of Chicago, and the first author of this article. In summary, data were generated from survey responses using the AmeriSpeak Panel. This panel, designed to be representative of the U.S. household population, had >54,000 members aged 13 and older within 43,000 households at the time of the survey in 2022 [5]. A total of 5837 panelists were invited to take the survey, of which 26.5% (1549 individuals) completed it. Sampling weights, provided by NORC, increased alignment with U.S. Census benchmarks for age, gender, education, race and ethnicity, and additional variables. More information on the development of the AmeriSpeak Panel, including its quality controls and weights, are described in the original data article.

Important to this updated data article, the newly released variables related to religious identity and religious service attendance were not a part of our March 2022 survey. Instead, they had been previously collected when respondents entered into the AmeriSpeak Panel with periodic updates by NORC. Thus, the timing of when the data were collected in these new variables matches that of the other sociodemographic data in this dataset, which were also previously collected by NORC. Although CoGenerate and the authorship team did not originally have access to these religious identity and religious service attendance variables because they were not in the original agreement with NORC, these variables were later shared (after publication of the original data article) due to a new data request. NORC provided permission to release these publicly.

## Limitations

The new data include a diverse set of respondents relating to religious identities. However, similar to the U.S. population overall, the sample is largely made up of Protestants, Catholics, other Christians, and non-religious individuals, making it difficult to conduct subgroup analysis within some religious identities (e.g., Jewish, Muslim, Hindu) due to small sample sizes.

## Ethics Statement

As stated in the original data article [4], NORC has an Institutional Review Board that is registered with the U.S. Department of Health and Human Services Office for Human Research Protections. The NORC IRB reviewed and approved this study protocol (Protocol ID: 22– 02– 662). Respondents, who provided informed consent to join the AmeriSpeak Panel, also agreed to take part in the survey that led to the creation of this dataset.

## CRediT Author Statement

**Cal J. Halvorsen:** Conceptualization, Formal analysis, Resources, Methodology, Writing – Original draft, Supervision. **Andrew Joung:** Formal analysis, Methodology, Writing – Reviewing and editing. **Stefanie Weiss:** Project administration, Writing – Reviewing and editing. **Jacob Stolmeier:** Data curation, Supervision, Project administration, Resources.

## Data Availability

DataverseCogeneration: A nationally representative survey of 1,549 Americans aged 18 to 94 on interest in, experience with, and barriers to working with older and younger people for social good (Original data).
